# Evaluation of Rhamnetin as an Inhibitor of the Pharmacological Effect of Secretory Phospholipase A2

**DOI:** 10.3390/molecules22091441

**Published:** 2017-08-31

**Authors:** Mariana Novo Belchor, Henrique Hessel Gaeta, Caroline Fabri Bittencourt Rodrigues, Caroline Ramos da Cruz Costa, Daniela de Oliveira Toyama, Luiz Felipe Domingues Passero, Marcia Dalastra Laurenti, Marcos Hikari Toyama

**Affiliations:** 1 Postgraduate Program in Food, Nutrition and Health, Federal University of São Paulo, Santos 11015-020, São Paulo, Brazil; belchor.mariana@gmail.com; 2 Bioscience Institute, Paulista State University, São Vicente 11330-900, São Paulo, Brazil; henriquehg@gmail.com (H.H.G.); cfabri3@gmail.com (C.F.B.R.); carolsbert@gmail.com (C.R.d.C.C.); felipepassero@yahoo.com.br (L.F.D.P.); 3 Pro-rector of Research, Brazil University, Itaquera 08230-030, São Paulo, Brazil; gaveira@yahoo.com.br; 4 Pathology Laboratory of Infectious Diseases (LIM50), Department of Pathology, Medical School of the University of São Paulo, São Paulo 01246-903, Brazil; mdlauren@usp.br (M.D.L.)

**Keywords:** rhamnetin, methylated quercetins, phospholipase A2, anti-inflammatory, *Bothrops jararacussu*

## Abstract

Rhamnetin (Rhm), 3-*O*-methylquercetin (3MQ), and Rhamnazin (Rhz) are methylated derivatives of quercetin commonly found in fruits and vegetables that possess antioxidant and anti-inflammatory properties. Phospholipase A2 (PLA2) displays several important roles during acute inflammation; therefore, this study aimed at investigating new compounds able to inhibit this enzyme, besides evaluating creatine kinase (CK) levels and citotoxicity. Methylated quercetins were compared with quercetin (Q) and were incubated with secretory PLA2 (sPLA2) from *Bothrops jararacussu* to determine their inhibitory activity. Cytotoxic studies were performed by using the J774 cell lineage incubated with quercertins. In vivo tests were performed with Swiss female mice to evaluate decreasing paw edema potential and compounds’ CK levels. Structural modifications on sPLA2 were made with circular dichroism (CD). Despite Q and Rhz showing greater enzymatic inhibitory potential, high CK was observed. Rhm exhibited sPLA2 inhibitory potential, no toxicity and, remarkably, it decreased CK levels. The presence of 3OH on the C-ring of Rhm may contribute to both its anti-inflammatory and enzymatic inhibition of sPLA2, and the methylation of ring A may provide the increase in cell viability and low CK level induced by sPLA2. These results showed that Rhm can be a candidate as a natural compound for the development of new anti-inflammatory drugs.

## 1. Introduction

Secretory phospholipases A2 (sPLA2) are proteins with a molecular mass of approximately 13 to 20 kDa. They are widely found in various animals, such as mammals, and in the venom of snakes, and they are almost exclusively calcium dependent. These enzymes have the same structural elements for enzymatic catalysis and have significant structural similarities among themselves [1,2,3,4]. sPLA2 and the calcium-dependent cytosolic PLA2 have the same enzyme triad, which allows these enzymes to hydrolyze membrane glycerophospholipids to produce arachidonic acid and lysophospholipids. Excessive hydrolysis of membrane phospholipids by activated sPLA2 may lead to altered membrane function, leading to functional membrane failure and cell death. In addition, free fatty acids and lysophospholipid hydrolysis products are precursors of bioactive pro-inflammatory mediators, such as eicosanoids and the platelet-activating factor (PAF) [4]. In particular, sPLA2 in snake venom is an attractive therapeutic target due to its accessibility, ease of purification and, in the case of sPLA2 in humans, high levels of systemic enzymatic activity, which characterize and contribute to most of the inflammatory disorders, including neurodegenerative diseases. Therefore, the discovery of new inhibitors of sPLA2 is essential to produce new safe and effective drugs with fewer side effects compared to the current therapy [5,6].

Flavonoids are a class of special metabolites from plants, characterized by the flavan nucleus. These compounds are widely distributed in the leaves, seeds, bark, and flowers of plants; in addition, a recent estimate showed that human’s intake ranges from 26 mg to 1 g per day [7]. Quercetin (Q) is a flavonol widely found in tea (*Camellia sinemensis*), wine, beer, fruits, and vegetables [8]. Additionally, this compound exhibits antioxidant and anti-inflammatory activities [9]. A methylated derivative of Q shows lipophilicity, which promotes the compound’s access to the cell. *Achyrocline satureioides*, *Coriandrum sativum*, and *Rhamnus petiolaris* are species that show the presence of 3-*O*-methylquercetin (3MQ), rhamnetin (Rhm), and rhamnazin (Rhz), respectively, and have anti-inflammatory activities. In all of these cases, there is no evidence of the effect of methylated quercetins, such as Rhm, against inflammation mediated by sPLA2 and the consequent mobilization of arachidonic acid metabolism or the possible side effects, including cytotoxic activities [10,11,12]. Considering the Q activities, this work aimed at evaluating 3MQ, Rhm, Rhz, and Q regarding this anti-inflammatory function, besides analyzing their cytotoxic activities toward the J774 cell lineage and evaluating the creatine kinase (CK) levels. In addition, the methylation of free hydroxyl groups in flavones results in more metabolically-stable derivatives with superior membrane-penetrating properties and, thus, vastly improves bioavailability, which should improve their ability to act inside cells [13]. The anti-inflammatory effects of Rhm by modulation of kinases, oxidative stress, and suppression of pro-inflammatory mediators have already been observed [11], however, there are no studies that reveal sPLA2’s inhibitory action of this compound and the relation of the structure and function with other quercetin derivatives. Thus, in this study, the inhibitory potential of Q and their methylated derivatives in vitro and in vivo were evaluated against sPLA2 from *Bothrops jararacussu*. To find safe and effective compounds, cytotoxic effects were evaluated using the cell lineage J774 as a model for cytotoxicity and CK levels were assayed as a model of acute toxicity in vivo.

## 2. Results

### 2.1. Purity of sPLA2

**Figure 1:**
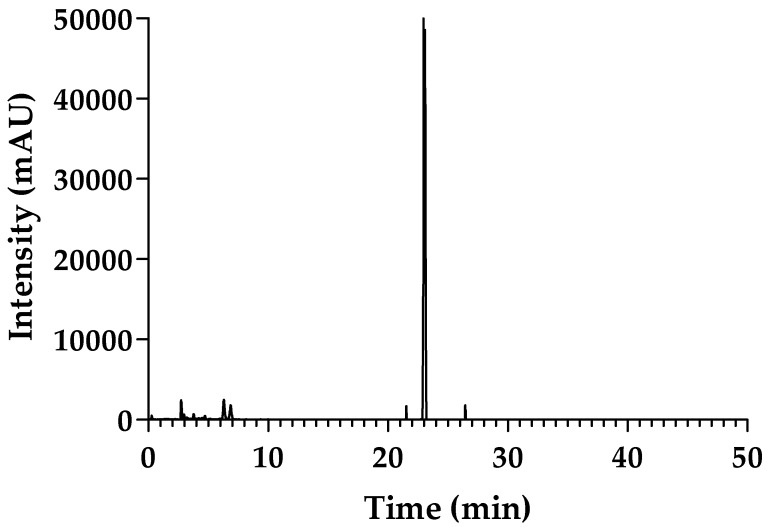
sPLA2 purity profile by high-performance liquid chromatography (HPLC). The enzyme purification was performed with a TSKgel SP-5PW (7.5 cm ID × 7.5 cm·L) using a non-linear gradient with buffer B (ammonium bicarbonate 1.0 M) at a constant flow rate of 1.0 mL/min. The active sPLA2 underwent a new chromatographic step on a reverse phase HPLC using a C5 semi-analytical column. In this step, the chromatographic column was pre-equilibrated with buffer A (0.1% TFA) for 30 min at a flow rate of 1 mL/min. Samples of sPLA2 (1 mg) were dissolved in 250 μL of buffer A and separated using the chromatographic column. Elution of the sPLA2 was performed with a continuous linear gradient of buffer B (66% acetonitrile in 0.1% TFA), and the monitoring of the chromatographic profile was at 280 nm.

sPLA2 purity was evaluated by high-performance liquid chromatography (HPLC). Figure 1 shows the chromatographic profile of the eluted native sPLA2 from *Bothrops jararacussu* with a retention time of 22.71 min.

### 2.2. Enzymatic, Circular Dichroism, Edema, and Myotoxic and Cytotoxic Effects

#### 2.2.1. Enzymatic Assay

**Figure 2:**
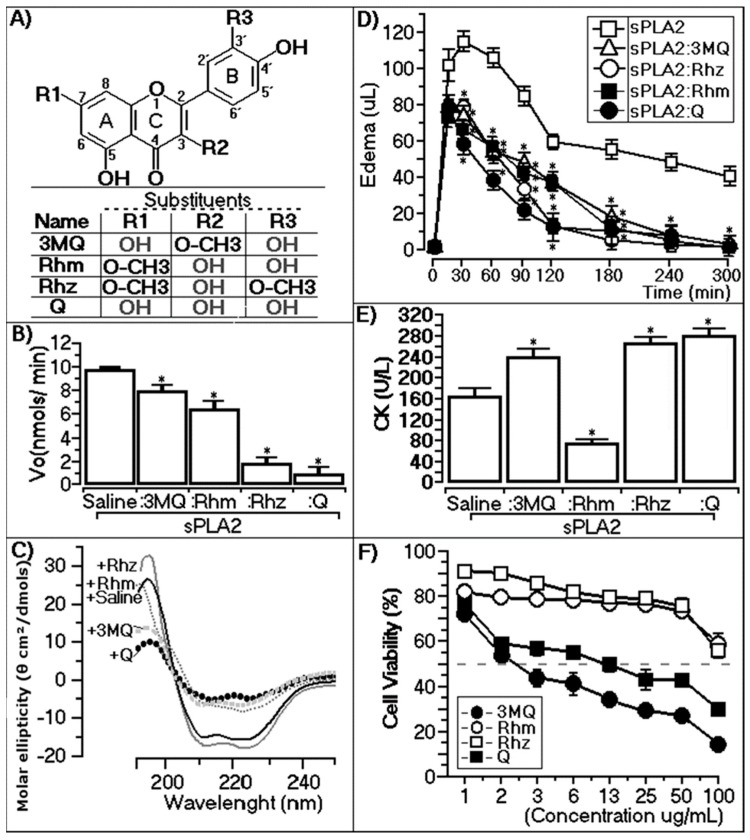
Structures of the quercetins assayed with the enzymatic, circular dichroism, edema, creatine kinase levels, and cytotoxic assays. (**A**) The structures of all flavonols assayed here and R1, R2, and R3 indicated the principal substituents found in all of the flavonols; (**B**) The effect of the previous treatment of the sPLA2 samples on the enzymatic activity after 30 min compared with native sPLA2. 3MQ, Rhm, Rhz, and Q. Each column represents the mean and SD of six replicates (*n* = 6), and the asterisk (*) means *p* < 0.05; (**C**) CD spectra of isolated sPLA2 (saline) and all quercetins incubated with the enzyme. Data over the range of 190–280 nm are shown and are expressed in theta machine units in molar ellipticity (θ cm²/dmols), and each spectrum represents the analysis of three CD runs; (**D**) Graphic of the edema performed after 30 min of administration of the four quercetins evaluated (10 μg protein and 12.5 μg compound). The results are expressed as the mean ± standard deviation (*n* = 5), and the * means *p* < 0.05. We used the ANOVA and Bonferroni a posteriori tests; (**E**) Myotoxic activity of native and pretreated sPLA2 via measurement of the creatine kinase assay. Each column is shown as the mean and SD of *n* = 5 for each sample including saline, and ANOVA was used as the statistic assay with Dunnett as the a posteriori test. The * shows groups with significant differences with sPLA2 (*p* < 0.05); (**F**) To evaluate the cytotoxic potential of all compounds, MTT (3-(4, 5-dimethylthiazolyl-2)-2,5-diphenyltetrazolium bromide) assay was performed with the J774 macrophages. Cells were incubated previously with each compound, and each point in the cell viability graph represents the mean and SD of 12 experiments.

All assays were conducted using sPLA2 from *Bothrops jararacussu* that was previously incubated with each compound (Figure 2A); under these conditions, 3MQ, Rhm, Rhz, and Q in the enzymatic assay revealed that, among the methylated quercetins, Rhz showed higher inhibition than 3MQ and Rhm. The inhibitory activity of Rhz was similar to Q. Despite the fact that Rhz showed methylation in the A ring and B ring, the presence of 3-OH in the C ring was likely a common group in Rhm, Rhz, and Q flavonols, leading to sPLA2 inhibition (Figure 1B). However, Rhz exhibits a higher inhibition than Rhm due the presence of the methylated group on the B ring. A greater interaction with sPLA2 was observed in Q, showing more inhibition among the quercetins studied. In addition, analysis of the results presented in Figure 2B,A showed that replacement of the OH group in the C ring by a methyl group almost completely abolished the inhibitory capacity of 3MQ.

#### 2.2.2. Circular Dichroism

Modifications in sPLA2 were evaluated via circular dichroism, and the effects in the α-helix and β-sheet are observable in Table 1. Figure 2C shows the changes as a result of the interactions of quercetins with sPLA2, which occurred mainly in the region corresponding to the α-helices. These results reveal the changes in the secondary structure of this protein due to the interaction with 3MQ, Rhm, Rhz, and Q (Figure 2C).

#### 2.2.3. Paw Edema Assay of Chemically-Treated sPLA2

All quercetins showed a significant inhibitor potential against sPLA2; thus, to evaluate the co-protection effect, the edema inhibition potential of the quercetins was evaluated. The compounds were administered 30 min before sPLA2 application. All flavonols studied for treatment of sPLA2 strongly decreased the maximum edema peak induced by native sPLA2 from *Bothrops jararacussu*. Moreover, Rhz and Q significantly diminished the edema induced by sPLA2 after 120 min. These results suggest that methylation substituents in the A and B ring and the presence of the 3-OH group in the C ring should play an effective role for the anti-inflammatory effect against edema induced by this native sPLA2 (Figure 2D).

#### 2.2.4. CK Levels of Chemically-Treated sPLA2

Named bothropstoxin II (BthTX-II), the PLA2 from *B. jararacussu* induced a rapid increase in plasma creatine kinase levels [14]. Thus, if compounds inhibit this enzyme, lower CK levels were observed, indicating less muscle damage. In this way, the CK levels were quantified to compare the compounds’ potential to decrease muscle injury. The results showed different activities for each compound. Q, 3MQ, and Rhz incubated with sPLA2 do not decrease the damage of this enzyme, and among these groups, CK levels were similar. By the other side, sPLA2 previously treated with Rhm potentially decreased CK release (Figure 2E). These data suggest that Rhm-treated sPLA2 neutralizes the muscle injury effect of native sPLA2 from *Bothrops jararacussu*. The structural analysis of all flavonols assayed here shows that the methylation in the A ring of Rhm is important for neutralization of the CK levels.

#### 2.2.5. Cytotoxic Assay of Methylated Quercetins

To evaluate the cytotoxic potential of all compounds, MTT assay was performed with the J774 cell lineage. Figure 1F shows the different cell responses of J774 cell lineage to quercetins. 3MQ and Q revealed a higher toxicity with CC_50_ = 3566 and 10,371 µg, respectively. Otherwise, Rhm and Rhz showed no toxicity toward J774 cell lineage. The analyses of these results suggest that replacement of the 3-OH group with the O-CH3 group in the C ring is involved with cytotoxic effects of 3MQ and Q (Figure 2D).

### 2.3. Edema Assay After Injection of sPLA2

**Figure 3:**
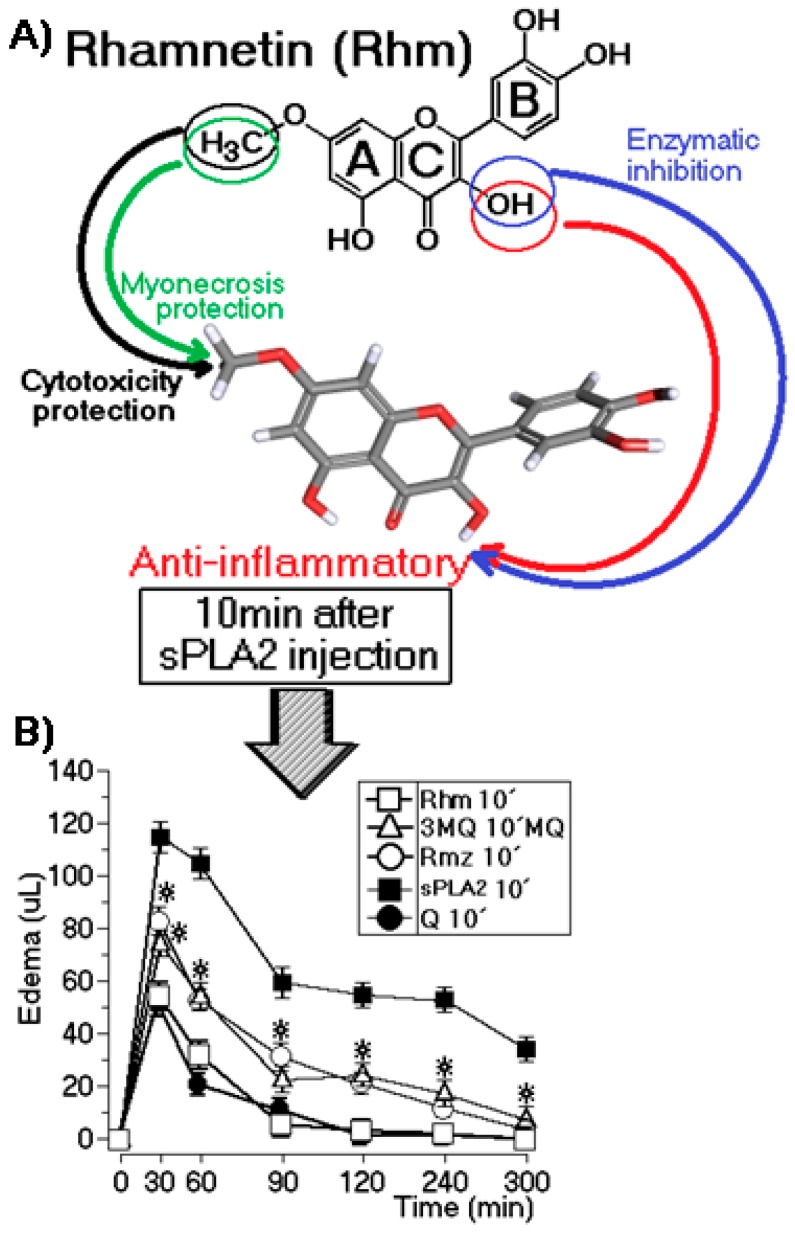
Structure-function relation between molecular regions of Rhm and its activities. (**A**) Molecular structure of Rhm in which the 3-OH in the C ring should lead to enzymatic and pharmacological inhibition and OCH_3_ in the A ring should lead to cytotoxic and myotoxic protection; (**B**) Flavonol injection after 10 min of sPLA2 application (10 μg protein and 12.5 μg compound). The results are expressed as the mean ± standard deviation (*n* = 5), and the * means *p* < 0.05 using the ANOVA and Bonferroni a posteriori tests.

In Figure 1, we summarize the effects of the chemical treatment of sPLA2 with 3MQ, Rhm, Rhz, and Q, and we concluded that Rhm has significant characteristics that include the presence of structural elements that specifically neutralize or strongly diminish the enzymatic activity of the pharmacological effects induced by sPLA2 from *Bothrops jararacussu* and do not induce significant cytotoxic activity. In the second edema assay, we injected each flavonol in the animals group 10 min after injection of sPLA2. Figure 3 shows that Rhm and Q essentially abolished edema induced by sPLA2, and both flavonols eliminated the edema 80 min after injection of sPLA2, whereas 3MQ and Rhz eliminated edema 4 h after injection of sPLA2.

## 3. Discussion

Plant extracts are characterized by the presence of active compounds and studies reveal the PLA2 inhibitory potential of extracts and isolated compounds. Crude aqueous extracts of *Casearia sylvestris*, *Piper umbellatum*, and *Piper peltatum* show this inhibitory activity [15,16,17,18,19,20]. Numerous studies have shown the anti-inflammatory and antioxidant activities of flavonoids and Q is widely distributed in vegetables and fruits [8,13]. Its potential and distribution highlight the search for new molecules and the need to understand their structures and functions. The methylation of polyphenolic compounds seems to be the second most significant conjugation reaction from a nutritional and metabolic point of view [21].

There are studies that have evaluated the effect of flavonoids and glycosylated flavonoids on the course of the enzymatic and pharmacological activity of secretory phospholipases A2 [5,6]. Moreover, these studies deserve further efforts to elucidate the mechanism of action of these types of flavonoids on sPLA2, including studies of the conformational changes of flavonoids that modify their antioxidant properties to pro-oxidants, and these mechanisms should be better understood. The chemical structure of polyphenols gives them the ability to act as free radical scavengers. The type of compound and the degree of methylation influence this capacity, and the number of hydroxyl groups is another parameter that determines the antioxidant activity [22]. Thus, within molecular logic, and with substantial data showing the antioxidant effects of various flavonoids, it would be assumed that any protective or neutralizing effect of flavonoids on sPLA2 could be associated with the presence of different numbers of OH moieties in the B ring of the flavonols. These characteristics may contribute to flavonoid’s antioxidant activity as well as its toxicity [23,24]. Despite the relevant role of sPLA2 in the course of the inflammatory process, there are not methylated flavonoid studies on the enzymatic and pharmacological activity of sPLA2. 

Results presented herein about the treatment of sPLA2 samples in the presence of Q and its derivatives clearly showed that it is not only the hydroxyls present in these flavonoids that are capable of neutralizing the pharmacological and enzymatic activities of sPLA2 from *Bothrops jararacussu*. The combination of methyl groups, the hydroxyl moiety present in the C-3 of the C ring and C-′4 of the B ring are shown to be essential to inhibit the enzymatic activity of sPLA2, as are the groups responsible for the neutralization of the edematogenic effects induced by this sPLA2 in this study. It can be accounted for because the C and B rings of some flavonoids may play a crucial role in the interaction of these compounds with the catalytic site of sPLA2 [5]; however, in the case of Rhm, the presence of the OH group in the C ring is crucial to decrease both the edematogenic and enzymatic activities of sPLA2. Besides that, the hydroxyl group present in the C-′4 also shows an enzymatic inhibitory role. In addition, our results showed that the methyl group present in the A ring of Rhm can be responsible to the absence of cytotoxicity and inhibition of high CK levels induced by sPLA2 from *B. jararacussu* venom. Once macrophages are essential in the primary response in inflammation [25], Rhm is revealed to be a compound that inhibits inflammation without impairing the viability of the body´s natural defence.

One important point is that the methylation of flavonoids may increase their hydrophobicity and, thus, increase the strength of the hydrophobic contacts between the protein and the flavonoid [26,27]. Studies with quercetrin reveal a high inhibition of sPLA2 from *Crotalus durissus terrificus*, possibly due the molecular interactions between the compound and the protein, as hydrogen bonding and hydrophobic interactions. The electrostatic interactions were observed between quercetrin and the amino acid residues Gly 30, Gly 32, His48, and Asp 49 and Ca^2+^ ion of sPLA2 leading to structural changes on the protein These interactions involves amino acids from de Ca^2+^-binding loop (e.g., Gly 30) and catalytic site (e.g., Asp 49 and His 48) [5]. Thus, besides the essential role of 3-OH to inhibit sPLA2, 4’-OH of Rhm can interact with Gly-30 of the protein leading to a higher decrease of its activity. These data suggest an essential role of hydroxyls on the interactions between the compound and sPLA2 and its following inhibition.

The circular dichroism clearly shows that, in the case of sPLA2, both methylations and the presence of hydroxyls can change the CD spectrum of sPLA2. Q strongly decreased sPLA2 α-helix and shows the higher enzymatic inhibition. Despite Rhz exhibit also a potent inhibition of the enzyme, CD data revels that this compound do not leads strong modifications in sPLA2, conserving the structure. High structure modification is observed in 3MQ, however, this change leads a decrease in the inhibition activity. Rhm interact with the enzyme besides exhibit an inhibitory potential. In this study, the presence of OCH3 on the catechol group increase sPLA2 inhibition, without strongly changing the enzyme structure. Despite the presence of OCH3 on the B ring, leading to a higher inhibition activity than Rhm, this methylation leads to high toxicity and high CK. Thus, 3-OH presence is also associated with sPLA2 inhibition, and methylation on the A ring brings no toxicity and lower CK. Higher interaction with sPLA2 of compounds with an absence of 3-OH was observed in 3MQ in this study and in quercetin [5]. These data are robust enough to show that methylation in the A ring is important to essentially abolish the cytotoxic and muscle damage effects via a free radical neutralizing-dependent pathway.

All flavonoids tested in this work are methylated derivatives of Q, which occurs naturally and is found in several species of phytochemical interest. In the case of Rhm, there is considerable evidence showing that this particular flavonoid exerts its anti-inflammatory activity through two basic mechanisms: via envelope suppression of free radicals and by pro-inflammatory mediators [27]. These results show that the myonecrosis induced by several basic sPLA2s, such as sPLA2 from this work, would involve a strong increase in oxidative cellular stress, and the antioxidant capacity of Rhm would be very interesting for treatment of muscular degeneration induced by this protein. In vivo, all quercetins showed co-protection effects, revealing a decrease in paw edema at all times. The co-protection effect of Q was elucidated in another study [28,29], and its effect on chronic inflammation was higher than hesperidin (glycosylated and methylated quercetin). As it is known, there is a greater potential for aglycone flavonoids in terms of antioxidant activity in relation to the glycosylated forms [28,29]. This lowest potential is due to the hydroxyl substitution at the C-3 position in the C ring with a methylated or glycosylated group as a result of a decrease in OH and the changes in the molecular conformation of hydroxyl group. The flavonols with OH in the C-3 position of the C ring are planar; thus, compounds are able to conjugate and relocate their electrons, as well as increase the radical phenoxyl stability of the flavonoid. Therefore, 3-OH absence results in a twist on the B ring changing the antioxidant potential [24,28,29]. In this study, 3MQ reveals a loss of its sPLA2 inhibition activity; it also shows high cytotoxicity and CK. These data suggest the essential role of methylations in the A ring, the planar conformation’s maintenance due to the presence of 3-OH and catechol group, besides the OH interactions between compound and protein to lead an inhibitory potential plus no toxicity against the immune system.

A decrease in paw edema was observed in *Cassia sophera* Linn, and this species has Rhm in its composition. In addition, *Coriandrum sativum* L. and *Prusmus cerasus* L. revealed the presence of Rhm, highlighting the potential of dietary species in phytochemical studies due to their benefits [11,12,30,31,32,33,34]. The suppression of the free radicals and pro-inflammatory mediators of Rhm would possibly inhibit of JNK and p38 MAPK activities by decreasing the COX2 pathway [11]. These pathways are important in the inflammation route, and our work shows that Rhm is beyond this activity; however, Rhm can both modulate and modify the action of sPLA2, thereby decreasing inflammation not only by maintaining inflammatory stimuli, but by also neutralizing enzymatic and pharmacological effects of sPLA2. In addition, in this work, Rhm and Q abolished the inflammatory effect induced by sPLA2 within 10 min after its injection—i.e., even after the onset of inflammatory process—Rhm and Q were effective for edema neutralization (Figure 2). However, the cytotoxicity results showed that the best choice is Rhm, and the fact that this flavonoid has molecular regions involved in the inactivation of sPLA2 (Figure 2), reveals that this compound inhibits the action of sPLA2 by neutralizing the protein, itself, and protecting the cell from the effects of free radicals. Studies reveal a protection function of catechol moiety of Q, improving cellular uptake, metabolic stability, and lower toxicity of the methylation in Q. Additionally, structural modifications at the 5 or 7 position preserve most of the antioxidant capacity and this feature is observed in Rhm, once methylation occurs at C-7 [35].

Studies reveal that nonsteroidal anti-inflammatory drugs (NSAIDs) are the most commonly used drugs in inflammatory diseases. However, these drugs inhibit cyclooxygenase (COX) and COX-1 inhibition leads to gastrointestinal and renal side effects [36]. The enzymatic reaction involved in prostaglandin production by activation of COX is a two-step reaction: cyclooxygenase catalyses the formation of PG from arachidonic acid and a subsequent peroxidase reduces hydroperoxides (PGG2) and activates the cyclooxygenase and the peroxidase activity of COX can also generate reactive oxygen species. Thus, there is no total certainty of inhibiting COX-2 without inhibiting COX-1 and there is still a third group of COX (COX-3), which are inhibited by the same COX-1 inhibitory drugs [37]. It is also important to remember that all COX-2 drugs target the activity of converting arachidonic acid to prostaglandin, but forget that COX is comprised of bifunctional enzymes, where the reactions of bis oxygenase and peroxidase occur at distinct sites, which are structurally and functionally interconnected. NSAIDs bind specifically to the arachidonic acid binding site, but do not inhibit the peroxidase site that remains active, and COX-1 is found in most tissues and is related to the production of prostanoids involved in homeostasis processes in the body [38]. Thus, by looking at the entire spectrum of COX-2, COX-1, and COX-3, the search for an inhibitory drug with inflammatory activity as a function of the specific inhibition of sPLA2 is complex, since the inhibition is not complete and the production of prostanoids is physiological, and COX-1 can also be modulated.

In turn, in this study it is believed that the structure and molecular conformation of Rhm has anti-inflammatory activity and did not induce toxicity to cell lineages, nor to the experimental model; therefore, it can be considered a prototype anti-inflammatory drug.

## 4. Materials and Methods

### 4.1. Flavonoids and Reagents

To understand the effects of flavonoids on edema, muscle damage and the cytotoxic effects of 3MQ, Rhm, Rhz, and Q, each of these were obtained commercially from Sigma-Aldrich (St. Louis, MO, USA) All other chemicals, reagents, and kits were purchased from Sigma-Aldrich^®^, Bio-Rad (Hercules, CA, USA), Cayman Chemical (Ann Arbor, MI, USA), and BIOMOL International (Farmingdale, NY, USA). 

### 4.2. Isolation, Enzymatic Kinetics, and Spectroscopic Evaluation of sPLA2

#### 4.2.1. Purification of sPLA2 from *Bothrops jararacussu*

The dried venom (35 mg) was dissolved in 0.05 M of ammonium bicarbonate buffer (buffer A) followed by centrifugation (4500× *g* for 3 min). The supernatant was studied via an HPLC system Jasco system) coupled to the TSKgel SP-5PW (7.5 cm ID × 7.5 cm·L) Tosoh Bioscience column (Minato-Ku, Japan). sPLA2 was collected using a non-linear gradient with buffer B (ammonium bicarbonate 1.0 M) at a constant flow rate of 1.0 mL/min. The chromatography was monitored at 280 nm, and the fraction obtained was lyophilized and stored at −20 °C. Once the phospholipase A2 enzyme activity of the corresponding fraction was confirmed, the active sPLA2 underwent a new chromatographic step on a reverse phase HPLC using a C5 semi-analytical column. In this chromatographic step, fractionation of sPLA2 was performed using C5 reverse phase chromatography, and the chromatographic column was pre-equilibrated with buffer A (0.1% TFA) for 30 min at a flow rate of 1 mL/min. Samples of sPLA2 (1 mg) were dissolved in 250 μL of buffer A and separated using the chromatographic column. Elution of the sPLA2 was performed with a continuous linear gradient of buffer B (66% Acetonitrile in 0.1% TFA), and the monitoring of the chromatographic profile was at 280 nm. 

#### 4.2.2. Enzymatic Characterization of sPLA2

The inhibitory potential of the quercetins against sPLA2 were observed. The enzyme was dissolved in a 0.9% NaCl saline solution at a final concentration of 1 mg/mL. sPLA2 samples were incubated with the quercetins in microplates for 20 min at room temperature in the presence of a chromogenic substrate for phospholipase (NOBA). The substrate was solubilized in acetonitrile at 1 mg/mL. To evaluate the inhibitory potential of the quercetins, sPLA2 was incubated with 0.25 mg/mL of the compounds for 30 min. Both were added to 0.02 M Tris-HCl, 0.15 M NaCl, and 1 mM CaCl_2_ (pH 8) buffer for analysis via a SPECTRA MAX spectrophotometer (Molecular Devices, Sunnyvale, CA, USA) at a wavelength of 405 nm. The measurements were performed throughout the incubation time (90 min) with five-minute intervals between readings. To calculate the percentage of inhibition, the rate of substrate consumption (slope of the line) was calculated using the formula

(1) $$\frac{\left(Vo_{PLA2}-Vo_{incubated}\right)}{Vo_{PLA2}}\times 100$$

The data were expressed in percentage, and the error is in percentage points.

#### 4.2.3. Circular Dichroism (CD)

J-815 CD Spectrometer (Jasco, São Paulo, Brazil) was used to evaluate the secondary structure of proteins (alpha helices, beta sheets, turns, and random secondary structures) in solution. It is characterized as a fast and economical method due to the small quantities of samples required. In this technique, polarized light is used in the distal ultraviolet (UV) range (from 180 to 260 nm). This technique allows the evaluation of the structural integrity of proteins, conformational changes, and processes of denaturation (unfolding) and renaturation (folding), making it possible to estimate the composition of the elements in the secondary structure of this macromolecule. The circular dichroism analysis was performed with 0.1 mg/mL of sPLA2, and the compounds were incubated at the same concentration (1:1). The isolated compounds were also used to evaluate the possible interference in the tests. The analysis was performed to eight convolutions, and data were treated by Spectra Manager.

### 4.3. Pharmacological Assay

#### 4.3.1. Paw Edema Evaluation

In vivo experimental models were performed to evaluate the inhibition of acute inflammation caused by purified sPLA2 using randomly-chosen Swiss female mice (~25 g, *n* = 5)*.* In vivo experiments were performed only after in vitro investigation of the inhibition or interaction activities of the compounds with sPLA2. Mice were injected with 50 μL (0.5 µg/g animal/12.5 µg per animal) of the compounds via peritoneal injection. After 30 min, via right posterior subplantar injection, 20 μL (10 µg) of PLA2 solution (*n* = 5) was inoculated. A saline solution (NaCl 0.9%) was used for negative control. Six groups were made: (1) Saline; (2) sPLA2; (3) sPLA2:3MQ; (4) sPLA2:Rhm; (5) sPLA2:Rhz; and (6) sPLA2:Q. The volume of the paws was monitored using the LE7500 Digital Plethysmometer (Panlab, Harvard Apparatus, Cornellà, Spain) for 4 h. After the tests, the mice were anaesthetized and sacrificed via cervical dislocation. In vivo experiments were performed according to the institutional rules and they were approved by the ethics committee from UNESP, number 008-CEUA.

#### 4.3.2. CK Level Measurement

In total, 20 μL (2.5 μg) of the compounds previously incubated for 30 min with sPLA2 (*n* = 5) were injected into the gastrocnemius muscle. After an average of 30 min, the animals’ blood was harvested from the tail in a heparinized tube, which was centrifuged and frozen for further analysis. Control groups (*n* = 5) were submitted to the same procedure as the treated animals but were inoculated with 20 μL of 0.9% NaCl, 20 μL (2.5 μg) of the purified compounds or 20 μL (10 μg) of isolated sPLA2. After the tests, the mice were sacrificed via cervical dislocation. Seric creatine kinase (CK) levels were determine the according to the manufacturer of the kit (Bioliquid, Pinhais, Brazil).

### 4.4. Cytotoxic Effects

To evaluate the cytotoxic effects of the quercetins, 2 × 10^5^ J774 cells were cultured in R10 medium with 3MQ, Rhm, Rhz, and Q (100.00 to 0.78 μg/mL). As a negative control, macrophages were cultivated in the medium and in DMSO as the vehicle solution (never exceeding 1% *v/v*). After 24 h, the cell viability was analysed via the MTT method. This cell lineage was employed in the present work as a model for cytotoxicity, as previously studied [39,40]. From a biological point of view, this cell lineage can be used as a prediction of the action of studied molecules on the immune system. Cytotoxic concentrations 50% (CC_50_) was estimated using GraphPad Prism 5.0 software (La Jolla, CA, USA) for plotting and statistical analysis.

### 4.5. Statistical Analyses

Data are expressed as the means ± standard deviations. The results were analyzed by analysis of variance (ANOVA) of one or two routes followed by the Dunett or Bonferroni a posteriori test. Values of *p* < 0.05 were considered significant.

## 5. Conclusions

Flavonoid methylations may be extremely interesting for the generation of structural and functional variability. In the case of Rhm, the methylation position on the Q skeleton gave rise to a compound that may be extremely valuable from a therapeutic point of view. This study shows that the methylation position changes the compound´s function and, among the evaluated quercetins, Rhm is the best choice, exhibiting sPLA2 inhibitory activity, no cytotoxicity, and lower CK levels.

## Figures and Tables

**Table 1:** Variation of the α-helix and β-sheet percentages in sPLA2 and in the quercetins incubated with the protein.

	sPLA2	3MQ	Rhm	Rhz	Q
α-helix	45.30%	26.80%	27.00%	54.70%	23.40%
β-sheet	14.80%	18.30%	18.00%	13.40%	18.80%

## Abbreviations

sPLA2	Secretory phospholipase A2
Q	Quercetin
3MQ	3-O-Methylquercetin
Rhm	Rhamnetin
Rhz	Rhamnazin
